# Pigtail Catheter: A Less Invasive Option for Pleural Drainage in Egyptian Patients with Recurrent Hepatic Hydrothorax

**DOI:** 10.1155/2016/4013052

**Published:** 2016-06-02

**Authors:** Mohamed Sharaf-Eldin, Adel Salah Bediwy, Abdelrahman Kobtan, Sherief Abd-Elsalam, Ferial El-Kalla, Loai Mansour, Walaa Elkhalawany, Mohamed Elhendawy, Samah Soliman

**Affiliations:** ^1^Tropical Medicine and Infectious Diseases Department, Faculty of Medicine, Tanta University, Tanta 35127, Egypt; ^2^Chest Diseases Department, Faculty of Medicine, Tanta University, Tanta 35127, Egypt

## Abstract

*Background and Aims*. Treatment of hepatic hydrothorax is a clinical challenge. Chest tube insertion for hepatic hydrothorax is associated with high complication rates. We assessed the use of pigtail catheter as a safe and practical method for treatment of recurrent hepatic hydrothorax as it had not been assessed before in a large series of patients.* Methods*. This study was conducted on 60 patients admitted to Tanta University Hospital, Egypt, suffering from recurrent hepatic hydrothorax. The site of pigtail catheter insertion was determined by ultrasound guidance under complete aseptic measures and proper local anesthesia. Insertion was done by pushing the trocar and catheter until reaching the pleural cavity and then the trocar was withdrawn gradually while inserting the catheter which was then connected to a collecting bag via a triple way valve.* Results*. The use of pigtail catheter was successful in pleural drainage in 48 (80%) patients with hepatic hydrothorax. Complications were few and included pain at the site of insertion in 12 (20%) patients, blockage of the catheter in only 2 (3.3%) patients, and rapid reaccumulation of fluid in 12 (20%) patients. Pleurodesis was performed on 38 patients with no recurrence of fluid within three months of observation.* Conclusions*. Pigtail catheter insertion is a practical method for treatment of recurrent hepatic hydrothorax with a low rate of complications. This trial is registered with ClinicalTrials.gov Identifier: NCT02119169.

## 1. Introduction

Treatment of hepatic hydrothorax is a clinical challenge, and patients with resistant hepatic hydrothorax often have few successful options [1].

Hepatic hydrothorax (HH) is defined as a transudative pleural effusion in patients with liver cirrhosis in the absence of cardiopulmonary disease. The estimated prevalence among patients with liver cirrhosis is approximately 5-6% [2].

HH is an infrequent but well-known complication of portal hypertension. Transdiaphragmatic passage of ascitic fluid from the peritoneal to the pleural cavity through numerous diaphragmatic defects has been shown to be the predominant mechanism in the formation of HH [3].

Patients with hepatic hydrothoraces often have few options [4]. Diuretic-resistant HH could be managed with liver transplantation, transjugular intrahepatic portosystemic shunt (TIPS), or indwelling pleural catheters. However, tube thoracotomy and pleurodesis have failed in many patients [5].

Small case series and a few case reports have recorded a high complication rate for insertion of chest tubes in cases of hepatic hydrothorax and have cautioned against their placement in hepatic hydrothorax. Chest tubes should not be placed in HH patients because high chest tube output and massive loss of fluid can lead to renal dysfunction and electrolyte disturbances. Because of the rapid reaccumulation of fluid in the pleural space as well as the high output, removal of the chest tube becomes difficult once it is placed [6, 7]. Pneumothorax, acute renal injury, and empyema were the most commonly reported. Death occurred in some of these cases. Insertion of chest tube for patients with hepatic hydrothorax has significant morbidity and even mortality, with questionable benefit [7].

The use of pigtail catheter for drainage of pleural effusion has proved to be safe and effective. So, it is recommended for any case of pleural effusion that requires drainage except for cases with loculated effusions that have low rate of success [8].

Treatment of hepatic hydrothorax is a clinical challenge. So, we assessed the use of pigtail catheter as a safe and practical method for treatment of recurrent hepatic hydrothorax as it had not been assessed before in a large series of patients.

## 2. Patients and Methods

The present case series study was conducted in Tanta University Hospital in Egypt. The Ethics Review Committee of the Faculty of Medicine, Tanta University, granted approval of this study, and informed consent was obtained from all the subjects.

The study included sixty cirrhotic patients admitted to the Department of Tropical Medicine, Tanta University Hospital, suffering from recurrent pleural effusion transudate in character. Light's criteria were applied (pleural fluid-to-serum protein ratio less than 0.5, pleural fluid lactic dehydrogenase (LDH) less than 200 IU, and pleural fluid-to-serum LDH ratio and pleural fluid-to-high normal serum LDH ratio less than 0.6).

Patients were excluded from the study if the character of the pleural effusion was exudative or they were suffering from hepatocellular carcinoma or other neoplasms, congestive heart failure, diabetes mellitus, impaired immune competence, a recent episode of digestive tract hemorrhage (i.e., within the previous 2 weeks), or ascitic fluid or pleural fluid infection.

Patients with platelet counts below 50,000 or prothrombin activity below 50% were also excluded.

All patients were submitted to diagnostic pleurocentesis within the first 48 hours of hospitalization and underwent complete evaluation including history taking, physical examination, chest X-ray PA and lateral views, liver function tests and renal function tests, complete blood count (CBC), prothrombin time and activity, abdominal ultrasonography, and pleural fluid and ascitic fluid analysis.

The degree of hepatocellular failure was evaluated by Child-Pugh classification and any complications were recorded. After hospital discharge, patients were followed up on an outpatient basis weekly for the first month and then monthly for 3 months. The follow-up included performance of complete liver and renal function tests in addition to recording any complications.

Some small case series and a few case reports have recorded a high complication rate for insertion of chest tubes in cases of hepatic hydrothorax, and there have been cautions against their placement in hepatic hydrothorax. Chest tubes should not be placed in HH patients because high chest tube output and massive loss of fluid can lead to renal dysfunction and electrolyte disturbances. Because of the rapid reaccumulation of fluid in the pleural space as well as the high output, removal of the chest tube becomes difficult once it is placed. We therefore thought it would be unethical to subject our patients to this dangerous maneuver and performed the study without a control group in whom chest tube placement was performed, preferring to compare our results to previous studies on chest tube placement.

## 3. Pigtail Catheter Insertion

We used pigtail catheter size 12 F with trocar. The site of catheter insertion was determined by ultrasound guidance and introduction performed under complete aseptic measures and proper local anesthesia. Needles were inserted just above the top of the rib to avoid injury of the intercostal bundle. A small (22-gauge) “finder needle” was employed before inserting the catheter. A small incision in the skin (usually of less than 5 mm) was made. Insertion was done by pushing the trocar and catheter until reaching the pleural cavity and then the trocar was withdrawn gradually while simultaneously introducing the catheter which was then connected to a collecting bag via a triple way valve.

Chest X-ray was done after the procedure to confirm the catheter being in place and to exclude any complications like pneumothorax.

Pigtail catheters were removed when drainage was less than 200 cc daily for 3 successive days. We considered the maneuver to be successful if the effusion disappeared on chest X-ray and if it was not required to have a second intervention (chest tube or surgery) within 3 days of catheter removal.

Patients received the standard therapy for liver impairment according to the hospital protocol and guidelines (e.g., diuretics and albumin infusion).

The end point of the study was either the resolution of the effusion and a decision to remove the catheter or the need for another intervention.

Pleurodesis was done for patients who had a successful procedure and accepted to have it performed. A mixture of 20 mL of 10% topical solution of povidone-iodine and 80 mL of normal saline and 2 mg/kg lidocaine 2% was instilled into the pleural cavity through the pigtail catheter and then the catheter was closed (via the triple way valve) for 6 hours. The patient's position was changed during this period by the medical staff to circulate the mixture. After opening the triple way valve, the catheter was kept in place until being removed when the fluid drainage volume was less than 100 cc per day.

Because of the possibility of systemic absorption of iodine (in povidone-iodine) and severity of thyroid disease, thyroid function testing was done before the performance of the procedure. Therefore, the patients with thyroid disease were excluded. Fortunately, none of our patients had abnormal thyroid functions or thyroid disease.

The study was approved by the Ethical Committee of the Faculty of Medicine, Tanta University, and the study was registered on ClinicalTrials.gov (ClinicalTrials.gov Identifier: NCT02119169).

## 4. Results

A total of 60 patients admitted to the Department of Tropical Medicine, Tanta University Hospital, who suffered from hepatic hydrothorax were enrolled in this study. They were 36 males (60%) and 24 females (40%). Their mean age was 42.3 ± 7.6 years.

Liver function tests and Child-Pugh score and pleural fluid characteristics for our patients in relation to protein, LDH, serum-pleural fluid albumin gradient, duration of drainage, and amount of drained fluid are presented in Table 1.

### 4.1. Complications of Pigtail Insertion

Complications were few and included pain at the site of insertion in 12 (20%) patients, blockage of the catheter in only 2 (3.3%) of the patients, and rapid reaccumulation of fluid in 12 (20%) patients. Complications of this maneuver are presented in Table 2.

#### 4.1.1. Pleurodesis

Pleurodesis was performed on 38 patients with no recurrence of fluid within three months of observation. Ten patients refused pleurodesis. In the remaining 12 patients, pleurodesis was not done because of rapid reaccumulation of fluid.

#### 4.1.2. Success Rate

The success rate of pigtail use in our patients was 80% (48 out of 60).

**Figure 1:**
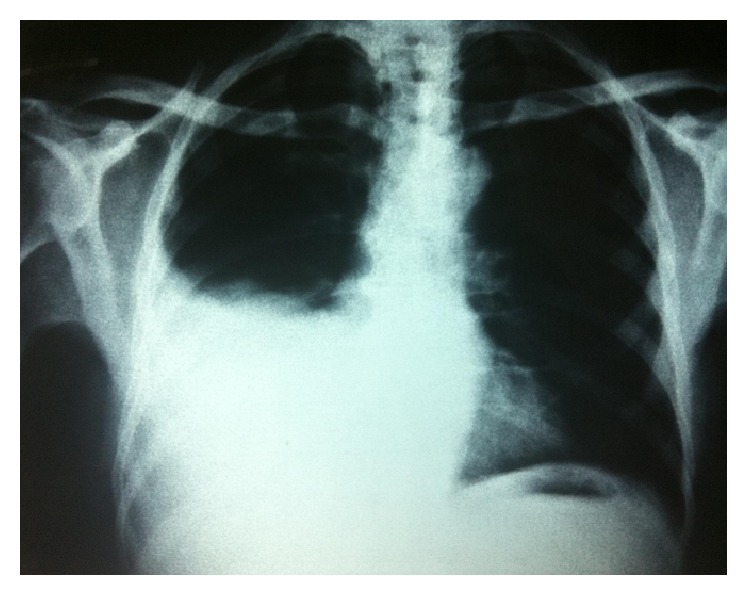
Chest X-ray showing right sided moderate pleural effusion (hepatic hydrothorax).

**Figure 2 fig2:**
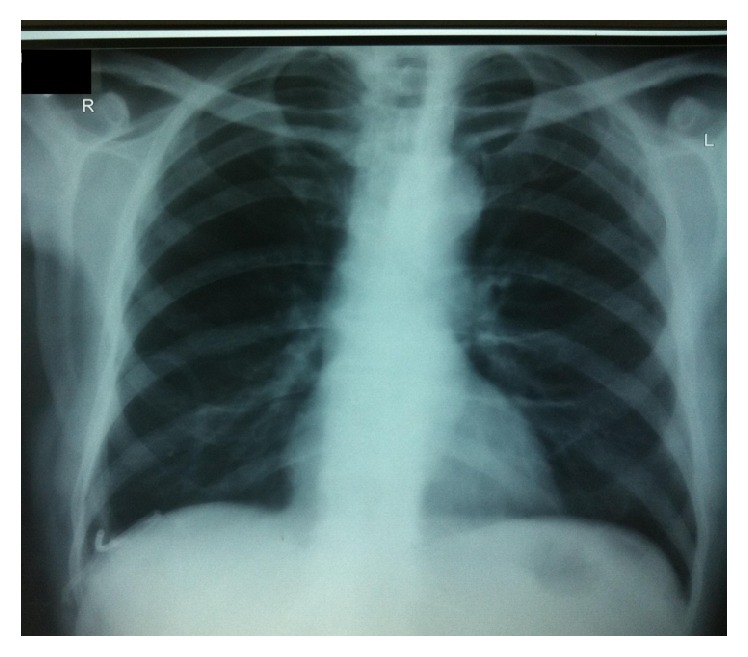
Chest X-ray of the same patient showing pigtail catheter in place with no effusion.

**Figure 3:**
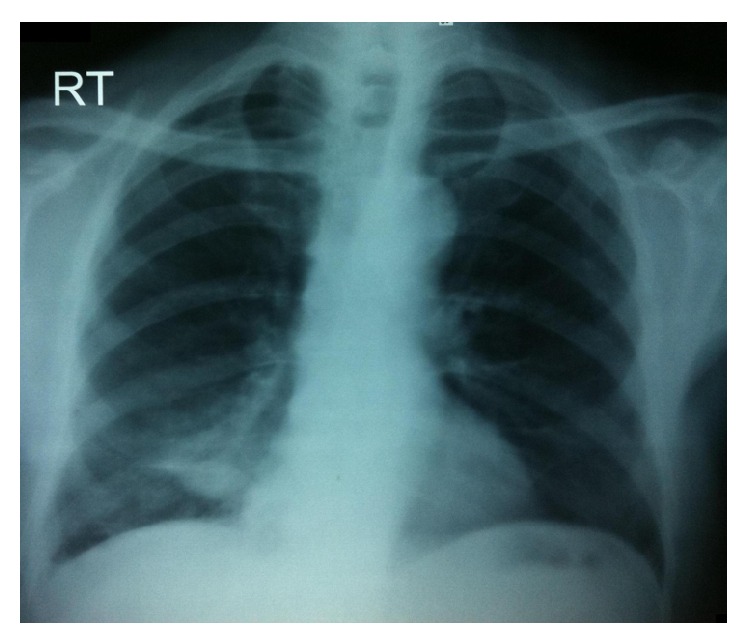
Chest X-ray of the same patient 2 months after pleurodesis and pigtail catheter removal showing no reaccumulation of fluid and successful pleurodesis.

Figures 1 [Fig fig2]–3 show X-rays of one of our patients before (Figure 1) and after (Figure 2) pigtail catheter insertion and 2 months following catheter removal and pleurodesis (Figure 3).

## 5. Discussion

Treatment of hepatic hydrothorax is a clinical challenge. We assessed the use of pigtail catheter as a safe and practical method for treatment of recurrent hepatic hydrothorax as it had not been assessed before in a large series of patients.

Our interest in the use of small-bore pigtail catheters for hepatic hydrothorax drainage was based on the concept that it is a less invasive measure and thus can be better tolerated by patients especially those with advanced liver diseases who cannot usually tolerate large chest tubes, with no decreased efficacy. Because of the high complication rate of chest tube insertion for hepatic hydrothorax, it is no longer recommended for drainage of such cases.

Drainage of pleural fluid decreased to less than 200 cc per day for 3 successive days in 48 patients giving them the chance to have a pleurodesis procedure. Among these 48 patients, 10 of them refused pleurodesis and the catheters were removed, while 38 patients had pleurodesis. Clinical improvement on success of the drainage was noted in the form of relief from dyspnea, cough, and hypoxaemia. Radiological improvement was in the form of absence of effusion from plain X-rays of the chest.

We found, in our study, that the duration of drainage of pleural fluid by using pigtail catheters was 10.3 ± 6.1 days. No previous studies on a similar number of patients with hepatic hydrothorax have been performed till now. However, the durations of drainage of pleural fluid of various other etiologies using a pigtail catheter were slightly shorter. For example, drainage period was 6 days (range from 3 to 21) in a study done by Parulekar et al. [9], 6.1 days reported by Liu et al. [10], and a range between 1 and 10 days recorded by Saffran et al. [11]. This may be attributed to compromised liver functions and marked hypoalbuminemia in patients with chronic liver diseases.

In the present study, complications were few and included pain at the site of insertion in 12 (20%) patients, blockage of the catheter in only 2 (3.3%) patients, and rapid reaccumulation of fluid in 12 (20%) patients. Pneumothorax did not occur in any of our patients and this was due to many factors: first, we did not use Seldinger technique as in our previous study [8] which usually needs a long time for insertion of the catheter allowing a chance for pneumothorax to occur; second, the large volume of pleural fluid producing high intrathoracic pressure in our patients prevented air from entering the pleural cavity during the maneuver; third, there was a high level of experience in performing the maneuver for many years at our centre.

Previous studies using pigtail catheters for pleural drainage revealed few complications. Only 5% of cases done by Roberts et al. [12] had serious complications like pneumothorax, hemothorax, and hepatic perforation. They found an overall complication rate of 20% of their cases including obstruction, dislodgement, empyema, disconnection, and kinking.

Another study done by Walsh et al. [13] found few complications of pigtail catheter use for pleural drainage. Pneumothorax (small, apical, and spontaneously resolving) occurred in 4 out of 15 patients. Only one patient developed reexpansion pulmonary edema.

Pleurodesis was performed using povidone-iodine, which is an effective, inexpensive, safe, and feasible agent for chemical pleurodesis previously used in the management of malignant pleural effusion [14, 15]. Among our 60 patients having pigtail catheter, pleurodesis was performed on 38 patients with no recurrence of fluid within three months. Ten patients refused pleurodesis and had successful pigtail drainage. In the remaining 12 patients, pleurodesis was not done because of rapid reaccumulation of fluid caused by the underlying hepatic condition which is the cause of pigtail catheter failure in these cases.

In our study, the success rate of pigtail use in our patients was 80% (48 out of 60). In all the six unsuccessful cases, failure was attributed to rapid fluid reaccumulation after pigtail catheter removal.

## 6. Conclusion

Pigtail catheter insertion based on our findings is an effective and safe way for pleural drainage in patients with recurrent hepatic hydrothorax and provides an excellent option for their treatment.

## Additional Points

(1) What is the current knowledge? (i) Treatment of hepatic hydrothorax is a clinical challenge. Chest tube insertion for hepatic hydrothorax is associated with a high complication rate, so newer techniques with fewer complications are needed. (2) What is new here? (i) We assessed the use of pigtail catheter as a safe and practical method for treatment of recurrent hepatic hydrothorax as it had not been assessed before in a large series of patients. (ii) Pigtail catheter insertion is a practical method for treatment of recurrent hepatic hydrothorax with a low rate of complications.

## Figures and Tables

**Table 1:** Liver function tests, child score, and pleural fluid characteristic of the studied patients.

*Liver functions*	
Serum bilirubin	2 ± 1.86 mg/dL
Serum albumin	2.7 ± 0.36 g/dL
AST	36.83 ± 22.77 U/L
ALT	55.06 ± 27.68 U/L
*Child-Pugh score*	
Child A	0 (0%)
Child B	32 (53.33%)
Child C	28 (46.66%)
*Pleural fluid characteristic*	
Protein	1.6 ± 0.4 gm%
LDH	103.5 ± 23.9 IU/L
Serum-pleural fluid albumin gradient	1.7 ± 0.3 gm%
Duration of drainage: mean ± SD	10.3 ± 6.1 days
Amount of drained fluid: mean ± SD	5.3 ± 1.7 liters

**Table 2:** Complications of pigtail catheter insertion.

Complications	Number of patients/percent
Pneumothorax	0/0%
Pain at site of insertion	12/20%
Blockage of the catheter	2/3.3%
Infection	0/0%
Rapid reaccumulation	12/20%
